# Ambulatory Assessment of Socio-Affective and Cognitive Aging: Zooming Into Daily Processes

**DOI:** 10.1093/geroni/igab046.2146

**Published:** 2021-12-17

**Authors:** Tabea Meier, Andrea Horn, Christina Roecke

## Abstract

Ambulatory assessment methods offer new possibilities to study cognitive, emotional, and social processes in the setting in which they naturally occur, namely in the daily life of individuals and couples. This allows to zoom into processes with great relevance for healthy aging and well-being over the lifespan. Research on daily psychological processes opens the door for investigating the interplay with contextual (daily stress, social resources) and stable factors (relationship quality status, accumulated discrimination) that are known to shape these dynamic processes. This symposium will present and discuss innovative contributions investigating daily emotional and cognitive functioning and their interplay with social and individual characteristics over the adult lifespan. The first study by Haas and colleagues will present experience sampling data on daily prospective memory performance in couples. Haas et al. will offer a dyadic perspective on cognitive functioning by examining how prospective memory performance is co-regulated in the daily lives of younger and older couples. Meier et al. will present another study from the same couples project; here the focus is on age differences in couples’ “we-ness” and its relationship with daily positive emotional experiences and how they are shared with the partner. The series of talks will be completed by Zavala et al. who examined associations between stress and emotional health in daily life and its interplay with prior discrimination experiences of an age-diverse sample of BIPOC adults. After these individual contributions, Christina Roecke will discuss the presented studies and provide her reflections on the results and their implications.

